# A Legendre tau-Spectral Method for Solving Time-Fractional Heat Equation with Nonlocal Conditions

**DOI:** 10.1155/2014/706296

**Published:** 2014-06-25

**Authors:** A. H. Bhrawy, M. A. Alghamdi

**Affiliations:** ^1^Department of Mathematics, Faculty of Science, King Abdulaziz University, Jeddah 21589, Saudi Arabia; ^2^Department of Mathematics, Faculty of Science, Beni Suef University, Beni-Suef 62511, Egypt

## Abstract

We develop the tau-spectral method to solve the time-fractional heat equation (T-FHE) with nonlocal condition. In order to achieve highly accurate solution of this problem, the operational matrix of fractional integration (described in the Riemann-Liouville sense) for shifted Legendre polynomials is investigated in conjunction with tau-spectral scheme and the Legendre operational polynomials are used as the base function. The main advantage in using the presented scheme is that it converts the T-FHE with nonlocal condition to a system of algebraic equations that simplifies the problem. For demonstrating the validity and applicability of the developed spectral scheme, two numerical examples are presented. The logarithmic graphs of the maximum absolute errors is presented to achieve the exponential convergence of the proposed method. Comparing between our spectral method and other methods ensures that our method is more accurate than those solved similar problem.

## 1. Introduction

In recent years, many engineering and physical phenomena can be successfully described by models of fractional differential equations (FDEs); see, for instance, [1–7]. Thus many researchers have been interested in studying the properties of fractional calculus and finding stable and robust numerical and analytical schemes for solving FDEs such as spectral tau method [8–10], Crank-Nicolson method [11], compact finite difference approximation [12], Legendre wavelets method [13], Haar wavelet operational matrix method [14], iterative Laplace transform method [15], Lie symmetry analysis method [16], and other methods [17–20].

Recently, spectral methods [21–23] have been applied to solve ordinary FDEs (see [24, 25]) while in [26, 27] the authors introduced the operational matrices of fractional derivatives with the help of the spectral methods to solve FDEs. This is not all; the partial FDEs are also investigated by using the spectral methods. In [28–31], the tau and collocation spectral methods are implemented in combination with the operational matrices of fractional integration for approximating the solution of some classes of space-fractional differential equations.

The T-FHE is a generalization of the classical heat equation obtained by replacing the first order time derivative by a fractional derivative of order *ν*, 0 < *ν* ≤ 1. Ali and Jassim [32] used the homotopy perturbation method to solve the T-FHE, while in [33] the authors introduced a general iteration formula of variational iteration method for a solution of the T-FHE. Moreover, in [34] the differential transform method is applied to solve the T-FHE. In addition, Rostamy and Karimi [35] constructed the Bernstein operational matrix for the fractional derivatives and used it together with spectral method to solve the T-FHE.

In this paper, we consider the T-FHE with the nonlocal condition [36]:

(1) $$\begin{array}{c} \frac{\partial^{\nu }u\left(x,t\right)}{\partial t^{\nu }}-\frac{\partial^{2}u\left(x,t\right)}{\partial x^{2}}=q\left(x,t\right),\;0<x\leq 1,\;\;0<t\leq 1, \end{array}$$

subject to

(2) $$\begin{array}{c} u\left(x,0\right)=u\left(x,1\right)+f\left(x\right),\;0<x\leq 1,\\ u\left(0,t\right)=g_{0}\left(t\right),\;u\left(1,t\right)=g_{1}\left(t\right),\;0<t\leq 1, \end{array}$$

where 0 < *ν* ≤ 1, *u*(*x*, *t*) is the temperature as a function of space *x* and time *t*, and *q*(*x*, *t*) is known source term. Our main aim is to achieve highly accurate solution of the T-FHE with nonlocal conditions (1) and (2). The tau-spectral method is applied based on the shifted Legendre polynomials as a basis function with the help of the operational matrix of fractional integration of such polynomials. Two numerical examples are introduced and solved using the presented technique to show its accuracy and validity. Also, we introduce comparisons between our numerical results and those obtained using the implicit difference approximation (IDA).

This paper is arranged in the following way: in Section 2 we introduce some definitions and notations of fractional calculus with some properties of Legendre polynomials. In Section 3 we apply our algorithm for the solution of the T-FHE with nonlocal condition. In Section 4 two numerical examples and comparisons between our results and those obtained by the IDA are introduced. Also in Section 5, a conclusion is presented.

## 2. Preliminaries and Notations

### 2.1. Fractional Calculus Definitions

Riemann-Liouville and Caputo fractional definitions are the two most used from other definitions of fractional derivatives which have been introduced recently.

**Definition 1 .**

The integral of order *γ* ≥ 0 (fractional) according to Riemann-Liouville is given by

(3) $$\begin{array}{c} I^{\nu }f\left(x\right)=\frac{1}{\Gamma \left(\nu \right)}\overset{x}{\underset{0}{\int }}{\left(x-t\right)}^{\nu -1}f\left(t\right)\;dt,\;\nu >0,\;\;x>0,\\ I^{0}f\left(x\right)=f\left(x\right), \end{array}$$

where

(4) $$\begin{array}{c} \Gamma \left(\nu \right)=\overset{\infty }{\underset{0}{\int }}x^{\nu -1}e^{-x}\;dx \end{array}$$

is gamma function.

The operator *I* ^*ν*^ satisfies the following properties:

(5) $$\begin{array}{c} I^{\nu }I^{\mu }f\left(x\right)=I^{\nu +\mu }f\left(x\right),\\ I^{\nu }I^{\mu }f\left(x\right)=I^{\mu }I^{\nu }f\left(x\right),\\ I^{\nu }x^{\beta }=\frac{\Gamma \left(\beta +1\right)}{\Gamma \left(\beta +1+\nu \right)}\;x^{\beta +\nu }. \end{array}$$

**Definition 2 .**

The Caputo fractional derivative of order *ν* is defined by

(6) Dνf(x)=1Γ(m−ν)∫0x(x−t)m−ν−1dmdtmf(t)dt,m−1<ν≤m,   x>0,

where *m* is the ceiling function of *ν*.

The operator *D* ^*γ*^ satisfies the following properties:

(7) $$\begin{array}{c} D^{\nu }C=0,\;\left(C\;\;\text{is}\;\;\text{constant}\right)\\ I^{\nu }D^{\nu }f\left(x\right)=f\left(x\right)-\overset{m-1}{\underset{i=0}{\sum }}f^{\left(i\right)}\left(0^{+}\right)\frac{x^{i}}{i!},\\ D^{\nu }x^{\beta }=\frac{\Gamma \left(\beta +1\right)}{\Gamma \left(\beta +1-\nu \right)}\;x^{\beta -\nu },\\ D^{\nu }\left(\lambda f\left(x\right)+\mu g\left(x\right)\right)=\lambda D^{\nu }f\left(x\right)+\mu D^{\nu }g\left(x\right). \end{array}$$

### 2.2. Shifted Legendre Polynomials

Assuming that the Legendre polynomial of degree *j* is denoted by *P*_*j*_(*z*) (defined on the interval (−1,1)), then *P*_*j*_(*z*) may be generated by the recurrence formulae

(8) $$\begin{array}{c} P_{j+1}\left(z\right)=\frac{2j+1}{j+1}zP_{j}\left(z\right)-\frac{j}{j+1}P_{j-1}\left(z\right),\;j=1,2,\ldots ,\\ P_{0}\left(z\right)=1,\;\;P_{1}\left(z\right)=z. \end{array}$$

Considering *z* = 2*x* − 1, Legendre polynomials are defined on the interval (0,1) that may be called shifted Legendre polynomials *P*_j_*(*x*) that were generated using the following recurrence formulae:

(9) Pj+1∗(x)=2j+1j+1(2x−1)Pj∗(x)−jj+1Pj−1∗(x),j=1,2,…,P0∗(x)=1,  P1∗(x)=2x−1.

The orthogonality relation is

(10) $$\begin{array}{c} \overset{1}{\underset{0}{\int }}P_{i}^{*}\left(x\right)P_{j}^{*}\left(x\right)dx=\{\begin{array}{cc} \frac{1}{2j+1},\; & \text{for}\;\;i=j,\\ 0,\; & \text{for}\;\;i\neq j.\; \end{array} \end{array}$$

The explicit analytical form of shifted Legendre polynomial *P*_j_*(*x*) of degree *j* may be written as

(11) $$\begin{array}{c} P_{j}^{*}\left(x\right)=\overset{j}{\underset{k=0}{\sum }}{\left(-1\right)}^{j+k}\frac{\left(j+k\right)!x^{k}}{\left(j-k\right)!{\left(k!\right)}^{2}}, \end{array}$$

and this in turn enables one to get

(12) $$\begin{array}{c} P_{j}^{*}\left(0\right)={\left(-1\right)}^{j},\;\;P_{j}^{*}\left(1\right)=1. \end{array}$$

Any square integrable function *u*(*x*) defined on the interval (0,1) may be expressed in terms of shifted Legendre polynomials as

(13) $$\begin{array}{c} u\left(x\right)=\overset{\infty }{\underset{j=0}{\sum }}a_{j}P_{j}^{*}\left(x\right), \end{array}$$

from which the coefficients *a*_*j*_ are given by

(14) $$\begin{array}{c} a_{j}=\left(2j+1\right)\overset{1}{\underset{0}{\int }}u\left(x\right)P_{j}^{*}\left(x\right)dx,\;j=0,1,\ldots . \end{array}$$

If we approximate *u*(*x*) by the first (*N* + 1)-terms, then we can write

(15) $$\begin{array}{c} u_{N}\left(x\right)=\overset{N}{\underset{j=0}{\sum }}a_{j}P_{j}^{*}\left(x\right), \end{array}$$

which alternatively may be written in the matrix form

(16) $$\begin{array}{c} u_{N}\left(x\right)≃A^{T}\Psi_{N}\left(x\right), \end{array}$$

with

(17) $$\begin{array}{c} A^{T}\equiv \left[a_{0},a_{1},\ldots ,a_{N}\right],\\ \Psi_{N}\left(x\right)\equiv {\left[P_{0}^{*}\left(x\right),P_{1}^{*}\left(x\right),\ldots ,P_{N}^{*}\left(x\right)\right]}^{T}. \end{array}$$

Similarly, let *u*(*x*, *t*) be an infinitely differentiable function defined on 0 < *x* ≤ 1 and 0 < *t* ≤ 1. Then it may be expressed as

(18) $$\begin{array}{c} u_{M,N}\left(x,t\right)≃\overset{M}{\underset{i=0}{\sum }}\;\overset{N}{\underset{j=0}{\sum }}u_{ij}P_{i}^{*}\left(t\right)P_{j}^{*}\left(x\right)=\Psi_{M}^{T}\left(t\right)U\Psi_{N}\left(x\right), \end{array}$$

with

(19) $$\begin{array}{c} U=\left(\begin{array}{cccc} u_{00} & u_{01} & \cdots & u_{0N}\\ u_{10} & u_{11} & \cdots & u_{1N}\\ \vdots & \vdots & \cdots & \vdots \\ u_{M0} & u_{M1} & \cdots & u_{MN} \end{array}\right), \end{array}$$

(20) uij=(2i+1)(2j+1)∬01u(x,t)Pi∗(t)Pj∗(x)dx dt,i=0,1,…,M,   j=0,1,…,N.

**Theorem 3 .**

The first derivative of the shifted Legendre vector Ψ_*N*_(*x*) may be expressed as

(21) $$\begin{array}{c} \frac{d\Psi_{N}\left(x\right)}{dx}=D\Psi_{N}\left(x\right), \end{array}$$

where **D** is the (*N* + 1) × (*N* + 1) operational matrix of derivative given by

(22) D=(dij)={2(2j+1),  for  j=i−k,   k=1,3,…,N,    if  N  is  odd,   k=1,3,…,N−1,    if  N  is  even,0, otherwise.

Repeated use of (21) enables one to write

(23) $$\begin{array}{c} \frac{d^{q}\Psi_{N}\left(x\right)}{dx^{q}}=D^{q}\Psi_{N}\left(x\right), \end{array}$$

where *q* is a natural number and **D** ^*q*^ means matrix power.

**Theorem 4 .**

The Riemann-Liouville fractional integral of order *ν* of the shifted Legendre polynomial vector Ψ_*M*_(*t*) is given by

(24) $$\begin{array}{c} I^{\nu }\Psi_{M}\left(t\right)=P_{\nu }\Psi_{M}\left(t\right), \end{array}$$

where **P**_*ν*_ is the (*M* + 1) × (*M* + 1) operational matrix of fractional integral of order *ν* and is defined by

(25) Pν=(∑k=00ξ(0,0,k)∑k=00ξ(0,1,k)⋯∑k=00ξ(0,M,k)∑k=01ξ(1,0,k)∑k=01ξ(1,1,k)⋯∑k=01ξ(1,M,k)⋮⋮⋯⋮∑k=0iξ(i,0,k)∑k=0iξ(i,1,k)⋯∑k=0iξ(i,M,k)⋮⋮⋯⋮∑k=0Mξ(M,0,k)∑k=0Mξ(M,1,k)⋯∑k=0Mξ(M,M,k)),

where

(26) ξ(i,j,k) =(2j+1)∑l=0j((−1)i+j+k+l(i+k)!(l+j)!       ×((i−k)!k!Γ(k+ν+1)(j−l)!         ×(l!)2(k+l+ν+1))−1).

(See [37] for proof.)

## 3. Legendre tau-Spectral Method

In this section, the Legendre operational matrix of fractional integrals is applied with the help of Legendre tau-spectral method to solve the T-FHE with the nonlocal condition.

Consider the T-FHE with the nonlocal condition

(27) ∂νu(x,t)∂tν−∂2u(x,t)∂x2=q(x,t),0<x≤1, 0<t≤1, 0<ν≤1,u(x,0)=u(x,1)+f(x), 0<x≤1,u(0,t)=g0(t), u(1,t)=g1(t), 0<t≤1.

We integrate (27) of order *ν* and making use of (7), we have

(28) u(x,t)−(u(x,1)+f(x))−Itν(∂2u(x,t)∂x2)  =Itνq(x,t), 0<ν≤1,u(0,t)=g0(t), u(1,t)=g1(t), 0<t≤1.

In order to use tau-spectral method based on the shifted Legendre operational matrix for fractional integrals to solve the fully integrated problem (28), we approximate (*x*, *t*), *f*(*x*), and *q*(*x*, *t*) by the shifted Legendre polynomials as

(29) $$\begin{array}{c} u_{M,N}\left(x,t\right)=\Psi_{M}^{T}\left(t\right)U\Psi_{N}\left(x\right),\\ f_{N}\left(x\right)=\Psi_{M}^{T}\left(t\right)F\Psi_{N}\left(x\right),\\ q_{M,N}\left(x,t\right)=\Psi_{M}^{T}\left(t\right)Q\Psi_{N}\left(x\right), \end{array}$$

where **U** is the unknown coefficients (*M* + 1) × (*N* + 1) matrix and **F** and **Q** are known matrices that can be written as

(30) $$\begin{array}{c} F=\left(\begin{array}{cccc} f_{0} & f_{1} & \cdots & f_{N}\\ 0 & 0 & \cdots & 0\\ \vdots & \vdots & \cdots & \vdots \\ 0 & 0 & \cdots & 0 \end{array}\right),\\ Q=\left(\begin{array}{cccc} q_{00} & q_{01} & \cdots & q_{0N}\\ q_{10} & q_{11} & \cdots & q_{1N}\\ \vdots & \vdots & \cdots & \vdots \\ q_{M0} & q_{M1} & \cdots & q_{MN} \end{array}\right), \end{array}$$

where *f*_*j*_ and *q*_*ij*_ are given as in (14) and (20), respectively.

Using (29), it is easy to write

(31) uM,N(x,1) =ΨMT(1)UΨN(x), =(∑k=0Muk0∑k=0Muk1⋯∑k=0MukN)ΨN(x), =ΨMT(t)VΨN(x),

where **V** is a (*M* + 1) × (*N* + 1) matrix that can be written as

(32) $$\begin{array}{c} V=\left(\begin{array}{cccc} \overset{M}{\underset{k=0}{\sum }}u_{k0} & \overset{M}{\underset{k=0}{\sum }}u_{k1} & \cdots & \overset{M}{\underset{k=0}{\sum }}u_{kN}\\ 0 & 0 & \cdots & 0\\ \vdots & \vdots & \cdots & \vdots \\ 0 & 0 & \cdots & 0 \end{array}\right). \end{array}$$

Making use of (23), (24), and (29) enables one to write

(33) Itν(∂2u(x,t)∂x2)≃(ItνΨMT(t))U(D2ΨN(x)),=ΨMT(t)PνTUD2ΨN(x).

In addition, if we use (24) and (29), we obtain

(34) $$\begin{array}{c} I_{t}^{\nu }q_{M,N}\left(x,t\right)=\Psi_{M}^{T}\left(t\right)P_{\nu }^{T}Q\Psi_{N}\left(x\right). \end{array}$$

Equations (31) and (34) enable one to write the residual *R*_*M*,*N*_(*x*, *t*) for (28) in the form

(35) $$\begin{array}{c} R_{M,N}\left(x,t\right)=\Psi_{M}^{T}\left(t\right)\left[U-V-F-P_{\nu }^{T}UD^{2}-P_{\nu }^{T}Q\right]\Psi_{N}\left(x\right). \end{array}$$

As in a typical tau method (see [22, 38, 39]) we generate (*M* + 1)(*N* − 1) linear algebraic equations in the unknown expansion coefficients, *u*_*ij*_, *i* = 0,1,…, *M*; *j* = 0,1,…, *N* − 2, namely;

(36) ∬01RM,N(x,t)Pi∗(t)Pj∗(x)dx dt=0,i=0,1,…,M, j=0,1,…,N−2,

and the rest of linear algebraic equations are obtained from the boundary conditions, as

(37) ΨMT(t)UΨN(0)=g0(ti),ΨMT(t)UΨN(1)=g1(ti),      i=0,1,…,M,

where *t*_*i*_, *i* = 0,1,…, *M* are the roots of *P*_*M*+1_*(*t*). The number of the unknown coefficients *u*_*ij*_ is equal to (*M* + 1)(*N* + 1) and can be obtained from (36) and (37). Consequently *u*_*M*,*N*_(*x*, *t*) given in (29) can be calculated.

## 4. Numerical Experiments

In order to highlight the accuracy of the presented scheme, we implement it to solve two numerical examples, and also comparisons between their exact solutions with the approximate solutions achieved using the presented scheme and with those achieved using other methods are made.

**Example 1 .**

We consider the following problem [36]:

(38) $$\begin{array}{c} \frac{\partial^{0.5}u\left(x,t\right)}{\partial t^{0.5}}-\frac{\partial^{2}u\left(x,t\right)}{\partial x^{2}}=\frac{2t^{1.5}\sin\left(2\pi x\right)}{\Gamma \left(1.5\right)}+4\pi^{2}t^{2}\sin\left(2\pi x\right),\;\\ u\left(x,0\right)=u\left(x,1\right)-\sin\left(2\pi x\right),\;0<x\leq 1,\\ u\left(0,t\right)=0,\;u\left(1,t\right)=0,\;0<t\leq 1, \end{array}$$

with exact solution *u*(*x*, *t*) = *t* ^2^sin(2*πx*).

Karatay et al. [36] introduced this problem and applied the IDA method to approximate its solution at various choices of time and space nodes *M* and *N*.

**Figure 1:**
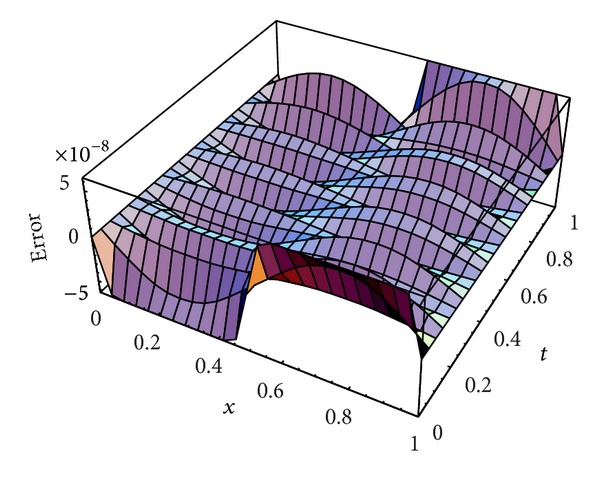
Absolute error function at *N* = *M* = 18 for Example 1.

**Figure 2:**
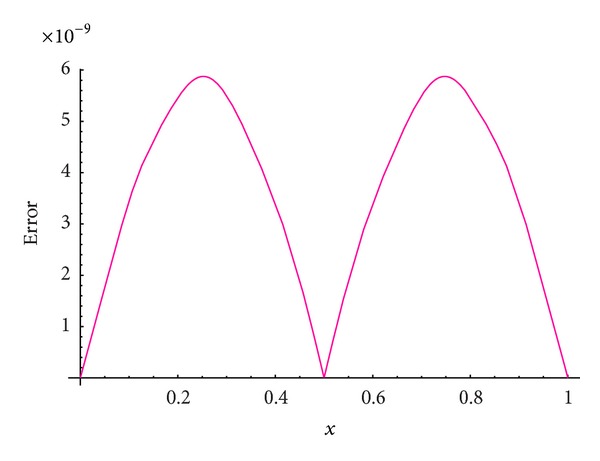
Absolute error function at *N* = *M* = 18 with *t* = 0.5 for Example 1.

We apply our numerical scheme for this problem. In order to show that our scheme is more accurate than the IDA method, in Table 1, we compare the maximum absolute errors (MAEs) achieved using our scheme with those obtained using the IDA [36] method at different values of *N*, (*N* = *M*). Moreover, Figure 1 plots the absolute error function at *M* = *N* = 18, while Figure 2 plots the absolute error function for *t* = 0.5 at *M* = *N* = 18.

**Example 2 .**

Consider the following problem:

(39) ∂νu(x,t)∂tν−∂2u(x,t)∂x2=2t2−νln⁡(1+x(1−x))Γ(3−ν)+t2(2x2−2x−1)(x2−x+1)2, 0<ν≤1,u(x,0)=u(x,1)−ln⁡(1+x(1−x)), 0<x≤1,u(0,t)=0, u(1,t)=0, 0<t≤1,

with exact solution *u*(*x*, *t*) = *t* ^2^ln⁡(1 + *x*(1 − *x*)).

**Figure 3:**
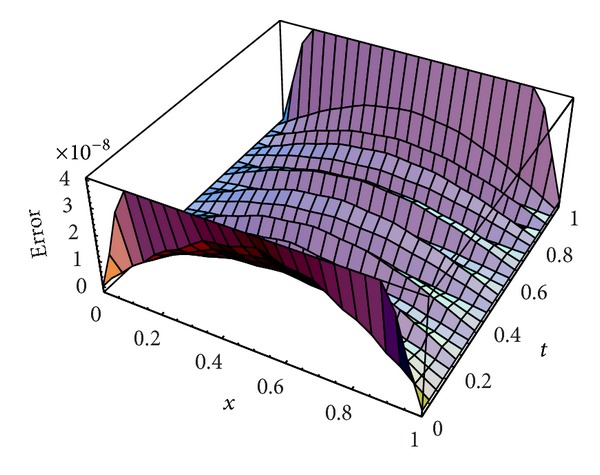
Absolute error function at *N* = *M* = 20 with *γ* = 0.45 for Example 2.

**Figure 4:**
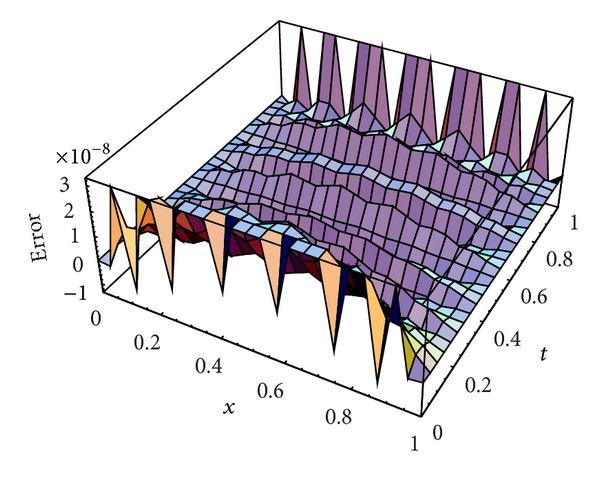
Absolute error function at *N* = *M* = 20 with *γ* = 0.95 for Example 2.

**Figure 5:**
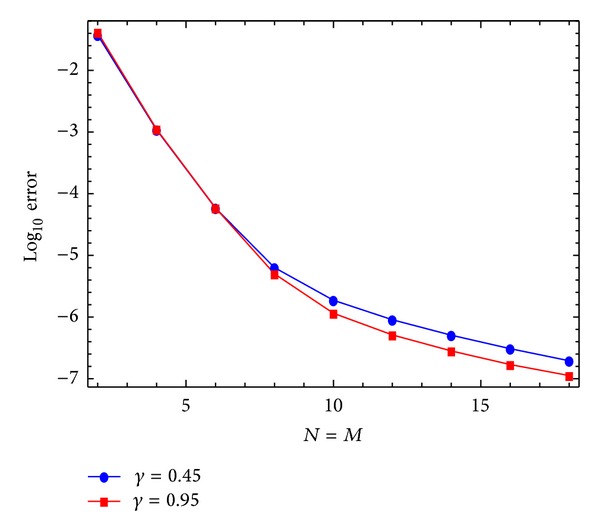
Absolute error function at *N* = *M* = 20 with *γ* = 0.95 for Example 2.

Karatay et al. [36] introduced this problem and solved it for two choices of *ν*, *ν* = 0.45,0.95 at different values of *N* and *M*.  Table 2 lists the MAEs for *ν* = 0.45, 0.95 using our scheme at *N* = *M* = 4,6, 8,10,12,14,16,18 and a comparison with those obtained in [36] at *M* = 16, *N* = 2,4, 8,16,32,64,128,256 and *N* = 16, *M* = 2,4, 8,16,32,64,128,256. Figures 3 and 4 plot the absolute error functions at *M* = *N* = 20 with *ν*_1_ = 0.45 and *ν* = 0.95, respectively. Finally, in order to demonstrate the convergence of the proposed method, in Figure 5, we plot the logarithmic graphs of the maximum absolute errors (log_10_Error) at two choices of *ν*, *ν* = 0.45, 0.95 and various choices of *N*, (*N* = *M*), by using the presented algorithm.

From Tables 1 and 2 and Figures 1 and 2 introduced above, it is shown that the proposed scheme is more accurate than the IDA method introduced by Karatay et al. [36].

## 5. Conclusion

An effective and accurate numerical scheme was developed to approximate the solution of the T-FHE with the nonlocal condition. The developed approach is based on the Legendre tau-spectral method combined with the operational matrix of fractional integration (described in the Riemann-Liouville sense) for orthogonal polynomials. A good approximation of the exact solution was achieved by using a limited number of the basis function.

The logarithmic graphs of the maximum absolute errors were presented to achieve the exponential convergence of the proposed method. Comparisons between our approximate solutions of test problems with their exact solutions and the approximate solutions achieved by the IDA method were introduced to confirm the validity and accuracy of our scheme.

## Figures and Tables

**Table 1:** Comparison of our scheme with the IDA [36] at various choices of *N*, (*N* = *M*) for Example 1.

Our scheme		IDA [36]	
*N* = *M*	MAEs	*N* = *M*	MAEs
6	2.541507 · 10^−2^	4	2.297695 · 10^−1^
8	8.774320 · 10^−4^	8	5.383793 · 10^−2^
10	2.118341 · 10^−5^	16	1.391800 · 10^−2^
12	1.832678 · 10^−6^	32	3.843610 · 10^−3^
14	1.037991 · 10^−6^	64	1.152111 · 10^−3^
16	6.590323 · 10^−7^	128	3.844224 · 10^−4^
18	4.383403 · 10^−7^	256	1.447756 · 10^−4^

**Table 2:** Comparison of our scheme with the IDA [36] at various choices of *N* and *M* for Example 2.

Our scheme			IDA [36]					
*N*	ν = 0.45	ν = 0.95	*M* = 16			*N* = 16		
			*N*	ν = 0.45	ν = 0.95	*M*	ν = 0.45	ν = 0.95
4	1.08 · 10^−3^	1.10 · 10^−3^	2	1.46 · 10^−2^	7.45 · 10^−3^	2	3.88 · 10^−2^	4.30 · 10^−2^
6	5.84 · 10^−5^	5.86 · 10^−5^	4	7.59 · 10^−3^	4.06 · 10^−3^	4	9.51 · 10^−3^	9.47 · 10^−3^
8	6.33 · 10^−6^	5.01 · 10^−6^	8	4.03 · 10^−3^	2.30 · 10^−3^	8	3.65 · 10^−3^	2.96 · 10^−3^
10	1.86 · 10^−6^	1.15 · 10^−6^	16	2.24 · 10^−3^	1.41 · 10^−3^	16	2.24 · 10^−3^	1.41 · 10^−3^
12	9.03 · 10^−7^	5.12 · 10^−7^	32	1.35 · 10^−3^	9.59 · 10^−4^	32	1.89 · 10^−3^	1.02 · 10^−3^
14	5.07 · 10^−7^	2.81 · 10^−7^	64	9.02 · 10^−4^	7.33 · 10^−4^	64	1.81 · 10^−3^	9.31 · 10^−4^
16	3.06 · 10^−7^	1.69 · 10^−7^	128	6.78 · 10^−4^	6.19 · 10^−4^	128	1.78 · 10^−3^	9.07 · 10^−4^
18	1.95 · 10^−7^	1.69 · 10^−7^	256	5.66 · 10^−4^	5.62 · 10^−4^	256	1.78 · 10^−3^	9.01 · 10^−4^
